# Traditional and Phytochemical Bases of Herbs, Shrubs, Climbers, and Trees from Ethiopia for Their Anticancer Response

**DOI:** 10.1155/2022/1589877

**Published:** 2022-02-03

**Authors:** Limenew Abate, Mesfin Getachew Tadesse, Archana Bachheti, Rakesh Kumar Bachheti

**Affiliations:** ^1^Department of Industrial Chemistry, College of Applied Science, Addis Ababa, P.O. Box-16417, Ethiopia; ^2^Centre of Excellence in Nanotechnology, Addis Ababa Science and Technology University, Addis Ababa, P.O. Box-16417, Ethiopia; ^3^Department of Environmental Science, Graphic Era University, Dehradun, Uttarakhand, India

## Abstract

Ethiopia is one of Africa's six plant-rich countries, with around 60% of the plants being indigenous and most of them having medicinal properties. 80% of people in the country use these plants as a primary health care system to tackle different diseases, including cancer. This review is aimed at summarizing the evidence gained from diverse MPs in Ethiopia that have been used ethnobotanically and ethnopharmacologically for treatment of cancer. The primary data sources were Google Scholar, Web of Science, Science Direct, Scopus, PubMed, and other electronic scientific databases. This literature review showed that there are around 200 MPs used as anticancer. Seventy-four herbs, 39 trees, 77 shrubs, and 17 weed/climbers belonging to 56 families have been identified for their ethnobotanical anticancer potential, and 31 species were recognized for their pharmaceutically anticancer activities. The reviewed data also indicated that many Ethiopian MPs had been used to treat breast, lung, blood, and skin cancers and other tumors. Besides, the collected data showed that the leaves (36.76%), roots (27.2%), bark (12.5%), stem (5.1%), and fruit (7.35%) of plants are commonly used for the preparation of anticancer remedies. Among the reported plant species, Euphorbiaceae (10.71%), Acanthaceae (7.14%), and Asteraceae (7.1%) are the most prominent plant families being used to treat cancer ethnobotanically. Phytochemicals such as flavonoids (like xanthone, indirubin, flavopiridol, and silybin), alkaloids (like taxol, vincristine, evodiamine, and berberine), and physalin B, D, and F steroids exhibited anticancer activity on various cancer cell lines. The crude extracts of *Aerva javanica*, *Vernonia leopoldi*, *Withania somnifera*, *Kniphofia foliosa*, and *Catharanthus roseus* were powerful anticancer agents with an IC_50_ value below 10 *μ*g/mL. Although several Ethiopian plants possess anticancer potential, only a limited number of plants are scientifically studied. Therefore, more scientific studies on anticancer MPs should be carried out; it may lead to discovering and isolating cost-effective and safe anticancer drugs.

## 1. Background

Plants are the sources of different chemical constituents such as essential oils, seed oils, and other phytochemicals, which gives a potential for various applications and pharmaceutical uses [1–4]. Specifically, traditional MPs (TMs) keep us healthy and treat different illnesses [5]. People have used TMs as primary health care contributors for thousands of years, and they play a vital role in preventing many diseases in resource-limited areas of developing countries [6, 7]. Nowadays, more attention has been given to TMs by different researchers because they can generate many uses and applications in the line of medicine and pharmacy [8]. In China, from total medicinal consumption, about 30% to 50% was gained from medicinal plants (MPs) [9]. In India, there are 17,500 native plant species from these 7,500 species that are used in ethnomedicines. About 2,000 aromatic and medicinal plant species are commercially used in Europe, while over 5,000 plant species are estimated to be used for medicinal purposes in Africa [10]. In Mali, Ghana, Nigeria, and Zambia, 60% of children's first treatment is obtained from a medicinal plant. In Ethiopia, approximately 80% of the population uses MPs because of the cultural suitability for local medicine [9].

Ethiopia has a vast diversity of plant species due to the presence of various topographical settings, ranging from the highest mountain to a deep valley; as a result, Ethiopia is rich in MPs [11]. There are about 6,000 plant species in the country, with 12 percent of them being endemic [12]. In Ethiopia, more than 800 plant species have been claimed to treat more than 300 ailments [13]. The bioactive compounds are responsible for the pharmaceutical properties of MPs [14] and can be isolated from plant seeds, fruits, bark, leaves, stems, roots, and flowers [15]. Alkaloids, terpenoids, flavonoids, glycosides, and polyphenols are bioactive compounds obtained from MPs and are used to cure various diseases, including cancer [16].

**Figure 1:**
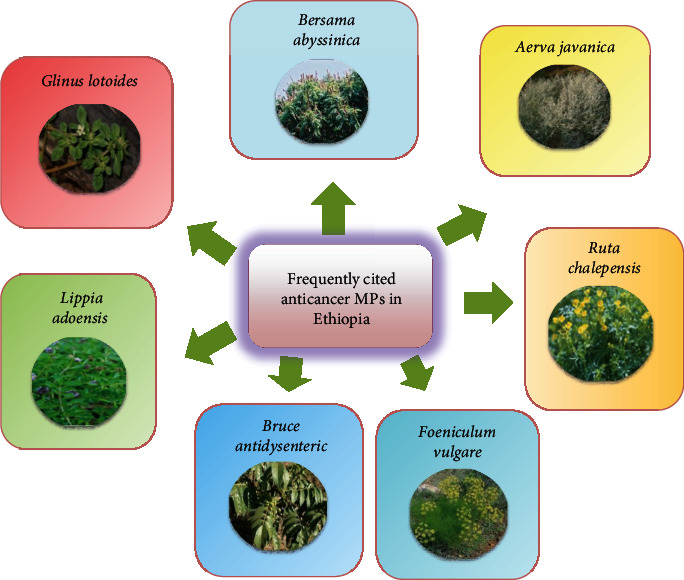
Frequently cited anticancer MPs found in Ethiopia.

Nowadays, cancer is one of the deadliest diseases in the world, which has been estimated to cause 9.9 million deaths in 2020 [17]. It also becomes a health problem in Ethiopia [18]. According to the report of Woldu et al. [19], each year, there are more than 150,000 cancer cases reported in the country; from these, about 4% result to deaths. The data obtained from the WHO indicated many types of cancers in Ethiopia; some of them are blood cancer, lung cancer, skin cancer, breast cancer, etc. [20]. Local people of Ethiopia have used different traditional practices to treat cancer [21]. MPs have been highly demanded in Ethiopia to treat cancer disease, because of their relatively low cost, the trust of communities on medicinal values of TMs being high, inadequate health centers, and shortage of drugs and personnel in clinics [13]. Some of the frequently cited anticancer MPs used by Ethiopian people to treat different types of cancers are *Aerva javanica*, *Bersama abyssinica*, *Asparagus africanus*, *Brucea antidysenterica* [22], *Nigella sativa* [23], *Matricaria chamomilla*, *Foeniculum vulgare* [24], *Zingiber officinale*, *Hibiscus sabdariffa*, *Glinus lotoides*, *Mentha piperita*, *Trachyspermum Ammi* [25], *Lepidium sativum* [26], *Commiphora myrrha* [27], *Ruta chalepensis* [28], and *Lippia adoensis* [29] as illustrated in Figure 1.

Although several Ethiopian plants were known to possess anticancer activity, very little scientific research is carried out on these MPs [30]. Also, a limited number of classes of secondary metabolites and pure isolated compounds were tested against cancer cell lines. Insufficient documentation on the ethnobotanical use of anticancer MPs is another problem in sharing traditional medicinal knowledge [31]. This review is aimed at giving an overview of the ethnomedicinal and phytochemical bases of anticancer MPs of Ethiopia.

## 2. Methods

Published research papers, review papers, proceedings, short communications, and book chapters on different MPs used to treat various forms of cancer in Ethiopia were retrieved from multiple databases such as PubMed, Web of Science, Scopus, and Google Scholar. More than 100 publications were obtained from 2007 to 2020. In the search process, keywords phytochemistry of anticancer plants of Ethiopia, traditional anticancer medicinal plant of Ethiopia, MPs used against cancer/tumor in Ethiopia, herbal medicine traditional medicine used against cancer in Ethiopia, and bioactive compounds isolated from the anticancer medicinal plant of Ethiopia were used. We classified the data according to the type of cancer (breast cancer, lung cancer, blood cancer, and skin cancer) and medicinal plant habits (shrub, herb, tree, weed, and climber). Additional important papers were also examined based on the reference list of the retrieved documents. ChemDraw was used to draw the structure of bioactive compounds, and pie charts were prepared using Excel software, while Endnote performed reference writing. We use the Natural Products Database for Africa (NDA) to write the botanical name and the local name of the medicinal plant.

## 3. Cancer Status in Ethiopia

Ethiopia is Africa's second-most populous country, and it is forecasted to become the world's ninth most populous country by 2050, with a projected increase in cancer burden [32]. Cancer is expected to account for around 5.8% of total national mortality in Ethiopia. Except for Addis Ababa, where population-based statistics are available, it is estimated that the annual incidence of cancer is about 60,960 cases and the annual mortality is over 44,000 [33]. According to a World Health Organization report on cancer [34], 77,352 new cancer cases were reported in both sexes of all ages, of which 26,754 were male of all ages and 50,598 were female of all ages. This showed that the number of new female cancer cases is 89.8% higher than that of males. Some of the recorded new cancer cases in 2020 were breast cancer (20.9%), leukemia (5.6%), cervix uteri (9.6%), colorectum (3.6%), and non-Hodgkin lymphoma (4.9%) (Table 1), and the mortality rates in the specified year were 24.1% (breast), 16.0% (cervix uteri), 3.9% (leukemia), 5.5% (ovary), 3.6% (lung), 3.4% (stomach), 5.5% (colorectum), and 5.9% (prostate) [34]. According to Tuasha et al. [11], from the total medicinal plant consumption used to treat cancer, 44.33% accounts for neck cancer, 14.0% breast cancer, and 10.67% skin cancer. The rest are the cancer of the brain, bone, rectal, lung, anus, cervix, and others.

### 3.1. Ethnobotanical Survey of MPs for Cancer Treatment

MPs are essential part of human life. For more than 2,000 years, they have been used as alternative medicine in the world [11]. Approximately 80% of these MPs globally are essential as the primary healthcare for fighting infections and treating illness [35]. MPs have been in continuous use over the years to manage cancer, particularly in most developing countries of the world [36]. The bioactive compounds of phytochemicals present in MPs are used to treat different diseases, including cancer [37]. For example, around 60% of drugs necessary for the cancer cure system have been derived from natural products of MPs [38]. Aromatic MPs are also crucial for medicinal purposes; they were considered the “father of medicine” by Hippocrates and ancient Greek physicians. Treating cancer and AIDS/HIV are their main benefits [21].

Many medicinal plant species found in Ethiopia are used to treat different types of illnesses for many years. Because the society believes in the therapeutic value of traditional medicines, of health center insufficiency, of the relatively low costs, of culturally related traditions, and of shortages of clinics and medical personnel, they are very popular in Ethiopia [13]. In Ethiopia, a large number of the human population (80%) and livestock (90%) directly or indirectly depend on traditional medicine [39]. According to a study conducted on traditional MPs in Ethiopia, the frequently cited diseases treated by these plants were cancers/tumors, stomach aches, wounds, coughs, headaches, skin diseases, toothaches, and diarrhoea [13]. Different studies on the ethnobotanical use of MPs from other parts of the country showed that traditional MPs are widely practiced to treat various cancer diseases such as lung cancer, breast cancer, and skin cancer [11]. Because of its ease of access and cultural acceptance, cancer patients choose traditional MPs for therapeutic approaches [40]. Ethnobotanical practices to treat cancer in Ethiopia are listed in Table 2.

**Figure 2:**
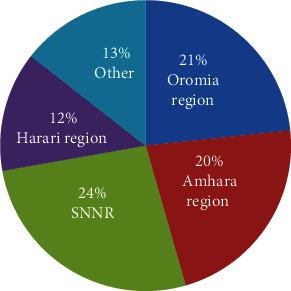
Region-wise distribution of anticancer MPs in Ethiopia.

**Figure 3:**
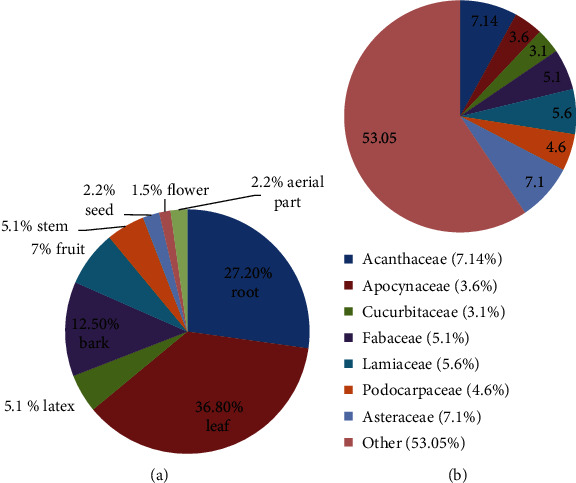
(a) Percent usage of different parts of MPs against cancer; (b) family-wise percentage of anticancer MPs.

Table 2 shows the list of 200 MPs which are used ethnobotanically against anticancer. Out of these, 33.8% are herbs, 17.9% trees, 39.5% shrubs, and 8.8% weed/climbers. Among the 56 families, Euphorbiaceae (10.71%), Acanthaceae (7.14%), and Asteraceae (7.1%) are the most prominent families which are used for ethnobotanical anticancer preparation. Regarding their distribution, 24% of MPs were found in Southern Nations, Nationalities, and People (SNNP), 21% in the Oromia region, and 20% in the Amhara regional state, as shown in Figure 2. The reviewed data also indicated that many Ethiopian MPs had been used to treat breast, lung, blood, and skin cancers. Plant sections that are widely used to make anticancer remedies were leaves (36.76%), roots (27.2%), bark (12.5%), stem (5.1%), and fruits (7.35%) (Figures 2 and 3).

### 3.2. Pharmacology Activities to Treat Cancer

#### 3.2.1. Plants Used against Breast Cancer

The most frequent cancer in women worldwide is breast cancer [37]. It is Ethiopia's most common cancer, with high morbidity and mortality rates. The number of new cases increases year to year in the country [71]. According to Memirie et al. [72], of all cancer cases in Ethiopia, 23% accounts for breast cancer. It accounts for 33% of the cancers in women. Breast cancer can be treated scientifically using different MPs. *Aerva javanica*, commonly known as “Tobia,” has been confirmed to be used for cancer care. The crude extract from the leaves of *Aerva javanica* has an antiproliferative effect on human breast cancer cell lines (MCF-7) [73]. *Kalanchoe petition*, commonly called “indahul,” used to cure breast cancer. The gallic acid isolated from the leave of *Kalanchoe petition* is responsible for its anticancer activity [56]. Extracts of *Sideroxylon oxyacanthum* are reported to be used frequently against breast cancer [49].

In another study, chloroform extract of aerial part of *Clematis simensis* was tested for anticancer activity using MTT assay against three breast cancer cell lines (JIMT-1, MCF-7, and MCF-10A). The IC_50_ (*μ*g/ml) values obtained after treating two breast cancer cell lines (JIMT-1 and MCF-7) and MCF-10A (one normal-like breast epithelial cell line) were as 80 ± 19, 190 ± 70, and 97 ± 9, respectively [74]. *Asparagus africanus*, named “Yeset-kest” in the local Ethiopian language, also treats cancer. The roots of the plant have been reported for treating breast tumors [22]. People of various religious and ethnic groups in Ethiopia use *Aerva javanica* as a traditional medicine to treat multiple diseases, including cancer. A scientifically validated study found that the leaf extracts of *Aerva javanica* showed an antiproliferative effect on human breast cancer cell lines (MCF-7) [42]. Alkaloids isolated from *Catharanthus roseus* showed potent cytotoxicity against the MDA-MB-231 breast cancer cell line, with IC_50_ values ranging from 0.97 ± 0.07 *μ*M to 7.93 ± 0.42 *μ*M [40]. In another work of Tesfaye and coworkers [75], they checked the cytotoxic activity of *Euphorbia schimperiana*, *Crambe abyssinica*, *Aloe debrana*, *Vachellia nilotica*, *Camellia sinensis*, *Termitomyces schimperi*, *Pentarrhinum insipidum*, *Acmella caulirhiza*, *Leonotis ocymifolia*, *Dorstenia barnimiana*, *Rumex nervosus*, *Clausena anisata*, *Helichrysum mannii*, *Salvia leucantha*, *Vernonia auriculifera*, *Corymbia brachycarpa*, and *Croton macrostachyus* extracts. Out of these, *Euphorbia schimperiana*, *Acokanthera schimperi*, *Kniphofia foliosa*, and *Kalanchoe petition* showed antiproliferative activity against human breast (MCF-7) cancer cell lines.

#### 3.2.2. Plants Used against Lung Cancer

Lung cancer is the leading cause of cancer-related deaths in men and the second leading cause of cancer-related deaths in women after breast cancer in the world [76]. GLOBOCAN 2020 is an online database providing global cancer statistics and estimates of incidence and mortality in 185 countries for 36 types and all cancer sites combined. According to GLOBOCAN data, there were approximately 18.1 million new cancer cases and 9.6 million deaths worldwide in 2018. Of these, 1.76 million died of lung cancer [77]. In the specified year, the number of new lung cancer cases in Ethiopia is 3.1% and it accounts for 4.3% of deaths from the total number of new cancer diseases [20]. Different MPs are used for the prevention and treatment of lung cancer. The seed extracts of *Glinus lotoides* (n-hexane, chloroform, methanol, and water) were tested for anticancer activity on the lung cancer cell line (Calu-3) using MTT assay. The result showed that methanol extract exhibits the highest anticancer activity with an IC_50_ value of 29.7 ± 1.3 *μ*g/mL, while water extracts (IC_50_ = 262.2 ± 1.2 *μ*g/mL) exhibit the least anticancer activity [78].

In another study, the anticancer activity of the root of *Aloe pirottae* was tested against stomach cancer (SNU-638), ovarian cancer (A2780), pancreatic cancer (MIA-PaCa-2), and lung cancer (A549) cell lines. The results demonstrated that all extracts exhibited anticancer activity with an IC_50_ value ranging from 6.37 to 29.69 *μ*g/mL [79].

The in vitro cytotoxic activity of essential oils and extracts of *Ocimum basilicum* was tested on a cancerous cell line (MCF-7). The result showed that the cytotoxic activity of essential oil was found to be more effective than that of the extracts [80].

Steroids extracted from *Withania somnifera* leaves were tested for the lung cancer cell line (NCI-H460). The result showed that steroids exhibited suitable anticancer activities with an IC_50_ value of 0.45 *μ*g/mL [81]. The cytotoxic activity T/Corr (%) of the extract (50 *μ*g/mL) on the lung cancer cell line A427 after 96 hours was tested by Tesfaye et al. [75], with a crystal violet cell proliferation test. According to their research result, *Crambe abyssinica*, *Aloe debrana*, and *Vachellia nilotica* showed values of 29.29, 49.65, 26.76, 26.41, and 46.62.

#### 3.2.3. Plants against Blood Cancer (Leukemia)

Blood-forming stem cells are the source of all blood cells. Blood cancer is caused by defects in the differentiation of these stem cells, which mainly affect white blood cells. Bone marrow transplantation, chemotherapy, antibodies, cytokines, and tumor vaccinations are choices for improving leukemia patients' survival rates [37]. Some Ethiopian plants such as *Clerodendrum myricoides*, *Myrsine melanophloeos*, and *Solanecio angulatus* have demonstrated anticancer activity in the case of leukemia [11]. The flower and leaf extracts of *Solanecio angulatus* were tested for anticancer activities against HL-60 human leukemia cell. The flower extract of the plant showed higher anticancer activities against the cell line with an IC_50_ value of 27.39 *μ*g/mL [82]. Essential oils of *Myrtus communis* were reported for the presence of 1,8-cineole, linalool, myrtenyl acetate, and myrtenol which is responsible for its anticancer activity against blood cancer (leukemia) [83]. Methanol and chloroform leaf extracts of *Cynoglossum coeruleum* were tested for anticancer activities against the HL-60 human leukemia cell line. The result indicated that the methanol extracts showed higher anticancer activity (IC_50_ = 183.95 *μ*g/mL) than chloroform extract (312.62 *μ*g/mL). The lowest IC_50_ value was recorded in methanol extract from *Cynoglossum coeruleum* flower with a value of 360.2 *μ*g/mL [82]. In another study, *Jatropha curcas* seed extracts displayed potent inhibition against P388 lymphocytic leukemia (both in vitro and in vivo) [42]. One research report showed that Alkaloids isolated from *Catharanthus roseus* such as vincristine, vinblastine, vindesine, vinorelbine, and vinflunine exhibited cytotoxic activity against human leukemia cells [84]. According to another study, the anticancer activity of crude extracts of *Rumex abyssinicus* roots was observed in prostate, brain, and breast tumor cell lines and leukemia cell culture [22]. Flavonoids, namely, alpinumisoflavone and 4′-methoxylicoflavanone extracted from *Erythrina asuberosa* stem bark, were tested for anticancer activity against HL-60 cells (human leukemia) and the result confirmed their anticancer activity [85].

#### 3.2.4. Plants Used against Skin Cancer

**Figure 4:**
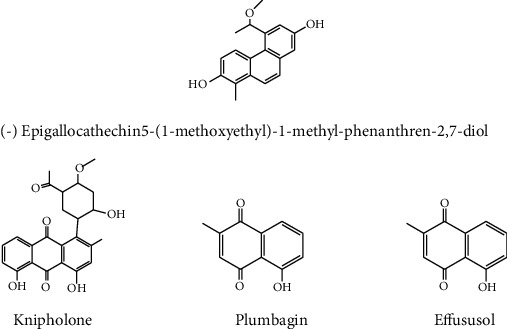
Anticancer phenolic compounds.

The most common cancer in the world is skin cancer. Melanoma is a type of skin cancer that involves basal and squamous cell carcinomas [37]. According to the WHO data from 2017, skin cancer deaths in Ethiopia accounted for 0.03 percent of all deaths. The age-adjusted death rate is 0.37 per 100,000 people of Ethiopia. The most recent WHO data from 2020 also showed that skin cancer deaths in Ethiopia accounted for 0.21 percent of all deaths, with new cases of 0.31% [86]. Phytochemicals with anti-inflammatory, immune-modulatory, and antioxidant properties have the best chance of acting as a chemopreventive in skin cancers [87]. Scopoletin (7-hydroxy-6-methoxy coumarin) from *Gelsemium sempervirens* has been reported to show anticancer activity against a skin cancer cell line (melanoma A-375) [88]. Plumbagin (a quinonoid constituent) (Figure 4) isolated from the root of *Plumbago zeylanica* was reported as having anticancer activity [89].

The methanol extract of the leaf of *Plantago lanceolata* was tested for anticancer activity. The result showed anticancer activities on the UACC-62 cell line with an IC_50_ value of 50.58 ± 11.15 *μ*g/mL[90]. Triterpenes found from the root of *Cucumis prophetarum* and gallic acid isolated from leaves of *Kalanchoe petitiana* are also used to cure skin cancer [90]. Bussa and Belayneh [21] reported the ethnomedicinal use of *Vernonia glaberrima* leaves and their phytoconstituents against skin cancer. The crude extract obtained from leaves, stems, and barks of *Clematis hirsute* is used for treating tumor/cancer on the neck [13].

## 4. Bioactive Compounds Used for Cancer Treatment

MPs are the source of many secondary metabolites known for their anticancer activity [37]. Phenolic compounds, alkaloids, glycosides, and terpenoids are some examples of such secondary metabolites with anticancer activity [30].

### 4.1. Phenolic Compound

In plant species, phenolic compounds are formed biologically via flavonoid, phenylpropanoid, and shikimate and possess hydroxide groups in the aromatic ring. These phenolic molecules have been shown for their cytotoxic, antiproliferative, and antioxidant characteristics [91]. Ethiopia has many MPs used to treat cancer; due to the existence of the phenolic molecule, for example, Okoye and coworkers [92] showed the anticancer activity of epigallocatechin extracted from *Maytenus senegalensis*. The bioactive compounds obtained from *Juncus effuses* such as 1-methylpyrene-2,7-diol, dehydrojuncusol, dehydroeffusol, effusol, effususol A, and 5-(1-methoxyethyl)-1-methyl-phenanthrene-2,7-diol (Figure 4) inhibited the proliferation of human cancer cell lines [93, 94]. Naphthoquinone isolated from *Plumbago zeylanica* extracts also treated human pancreatic and lung cancers [95, 96]. In another study, isolated compounds knipholone and knipholoneanthrone from *Kniphofia foliosa* were tested for anticancer activity against leukemic and melanocyte cancer cell lines. The results indicated that knipholoneanthrone has a potential anticancer agent [97].

According to a study on the biological activities and phenolic compounds of ethanolic extracts from *Zingiber officinale* and *Curcuma longa* rhizomes, the plants have anticancer properties in the B164A5 murine melanoma cell line due to the presence of phenolic compounds [98].

### 4.2. Flavonoids

Flavonoids are polyphenolic compounds that make up a broad family of secondary metabolites found in plants [85]. Various research showed that flavonoids in different plants had been used for anticancer activities [99]. Multiple studies have shown that increasing the number of flavonoids in one's diet will reduce cancer risk [100]. Quercetin, chalcones, genistein, curcumin, isoflavones, flavanones, and cisplatin are used to treat human oral cancer while daidzein, genistein, quercetin, luteolin, and flavanones are used to treat human breast cancer. Human lung cancer can be treated with flavone and quercetin [101]. Some flavonols like epicatechin, catechin-3-gallate, epigallocatechin flavan-4-ols, flavan-3, 4-diols, flavan-3-ols, catechin, and gallocatechin also are used to treat different cancers such as prostate and rectal cancers. Flavones such as luteolin, chrysin, apigenin, flavonol: rutin, quercetin, myricetin, kaempferol flavanones: naringenin, hesperidin, eriodictyol, flavanonols: taxifolin are used to take care of lung cancer, laryngeal cancer, and breast cancer [102].

In human leukemia cells, flavonoids extracted from *Erythrina suberosa* stem bark such as 4′-methoxylicoflavanone and alpinumisoflavone were found to have cytotoxic effects [85]. Flavonoids extracted from *Cassia Angustifolia*, such as scutellarein, quercimeritrin, and rutin demonstrated considerable anticancer activity against MCF-7, Hep2, and HeLa cell lines, with lower cytotoxicity towards the HCEC cell line [103]. The crude extracts/fractions of *Clerodendrum myricoides*, *Vernonia leopoldi*, *Dovyalis abyssinica*, *Sideroxylon oxyacanthum*, *Clematis longicauda*, *Zanthoxylum chalybeum*, and *Clematis simensis* were tested for anticancer activities and found cytotoxic effects against various breast cancer-derived cell lines [74].

**Figure 5:**
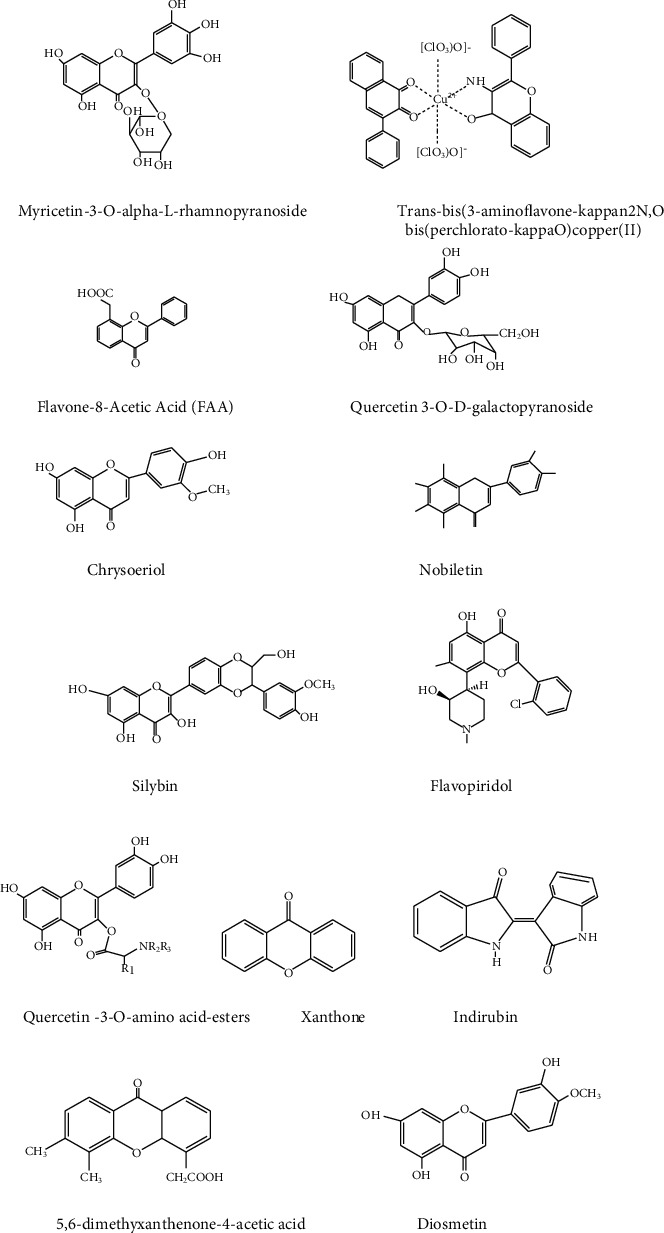
Chemical structure of anticancer flavonoids.

Bioactive compounds such as luteolin, sesquiterpene lactones, coumarins, and phenolic acids isolated from leaves and shoots of *Vernonia amygdalina* have shown cancer chemoprevention [44]. One study observed the anticancer activity in *Cassia angustifolia* extract seed powder against the tested HCEC, Hep2, HeLa, and MCF-7 cell lines. The IC_50_ value of methanol extract against HeLa cells was 5.45 g/L and 4 g/L against MCF-7 cells, lower than the drug taxol 6.07 g/L and tamoxifen 6.4 g/L. This anticancer characteristic is due to bioactive flavonoids such as quercimeritrin, scutellarein, and rutin in the plant's seed [104]. The different ethanolic extracts of *Lagenaria siceraria* were studied for anticancer activity against MCF-7. The result confirmed that it inhibits cancer cells in a concentration-dependent manner with a maximum concentration of 80 *μ*g/mL. This anticancer activity of the extract can be attributed to its flavonoid and polyphenol contents in the extracts [55]. Some of the advanced anticancer flavonoids used to treat cancers are myricetin-3-O alpha-L-rhamnopyranoside, flavone-8-acetic acid, quercetin 3-O-D galactopyranoside, chrysoeriol, nobiletin, silybin, flavopiridol, quercetin-3-O-amino acid-esters, xanthone, indirubin, 5,6 dimethylxanthenone-4-acetic acid, diosmetin, and myricetin-3-O-alpha-L-rhamnopyranoside (Figure 5) [101].

### 4.3. Alkaloids

**Figure 6:**
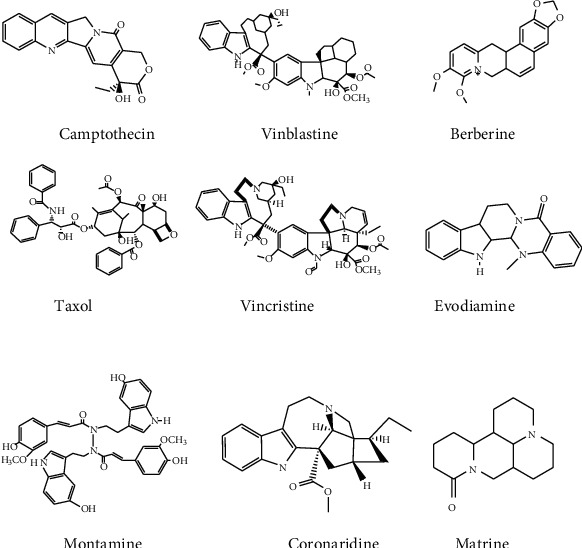
Structure of some anticancer alkaloids.

Alkaloids are essential chemical compounds that can be used to discover new drugs. In vitro and in vivo, some alkaloids derived from natural herbs have antimetastasis and antiproliferative effects on various cancers. Alkaloids including vinblastine and camptothecin have also been used to develop anticancer drugs [104]. The vinca alkaloids, such as vinblastine, vinorelbine, and vincristine, were the first plant-derived anticancer agents to gain approval for clinical use [87]. Some of the alkaloids used having anticancer activities are taxol, vincristine, vinblastine, 9-methoxycamptothecin, berberine, schischkiniin, coronaridine, naucleaorals, monoamine, camptothecin, an indole alkaloid, and protoberberine [105] (Figure 6). In Ethiopia, the alkaloids extracted from the root of *Gloriosa superba* are used to treat breast cancer. When the root is chewed and applied externally to the affected area, it relieves and recovers pain [22, 42]. Phytochemical studies conducted in the Harari region have shown that the alkaloids and glycosides in the roots of *Hydnora abyssinica* are vital for cancer treatment [38]. The chloroform extract of *Clematis simensis*, rich in alkaloid bioactive compounds, showed cytotoxicity against three breast cancer cell lines. Two breast cancer cell lines JIMT-1 and MCF-7 showed IC_50_ values of 80 *μ*g/mL and 190 *μ*g/mL, respectively. One of the normal-like breast epithelial cell lines (MCF-10A) has 97 *μ*g/mL [74]. The alkaloids, which are also present in the flower of *Solanecio angulatus*, showed in vitro cytotoxicity properties with an IC_50_ value of 133.72 *μ*g/mL in the tested cell line (HL-60) [30]. Solasonine and solamargine alkaloid (Figure 6) molecules, which were isolated from *Solanum nigrum*, exhibited anticancer activities on the human gastric cancer cell line (MGC-803) with IC_50_ values of 5.2 *μ*g/mL and 8.77 *μ*g/mL, respectively [40].

### 4.4. Steroids

**Figure 7:**
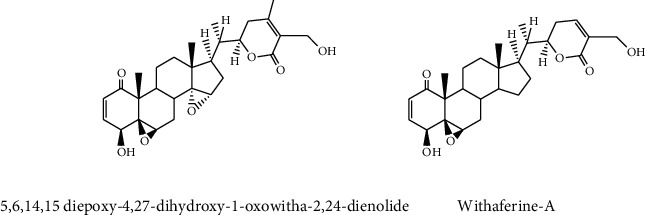
Structure of anticancer steroids.

A group of natural or synthetic organic compounds with a molecular structure of 17 carbon atoms grouped in four rings is known as steroids. In genetics, chemistry, and medicine, steroid hormones play a significant role. Hundreds of steroids have been discovered in fungi, animals, and plants [106]. Medicinal plant steroids are well-known secondary metabolites to have anticancer activity [107]. Bioactive compounds of steroids which were isolated from *Withania somnifera* such as 5,6,14,15 diepoxy-4,27-dihydroxy-1-oxowitha-2,24-dienolide and withaferin-A (Figure 7) showed anticancer activity to the human lung cancer cell line (NCI-H460) with 0.45 *μ*g/mL and 8.3 *μ*g/mL IC_50_ values, respectively, [81]. In addition, cytotoxic activities were shown in extracts of *Bersama abyssinica.* Hellebrigenin 3,5 diacetate, hellebrigenin 3-acetate, bersenogenin, 3-epiberscillogenin, and berscillogenin demonstrated cytotoxic activities in the plant extract [108, 109]. Physalin B, D, F steroids which are found in *Physalis angulate* showed anticancer activities on different cancer cell lines such as KB, A549, HCT8, and PC3 with the lowest EC_50_ (*μ*g/mL) value of 0.9 (for KB), 1.3 (for A549), 1.0 (for HCT8), and 0.9 (for PC3), respectively, for physalin F, physalin D, and physalin B [110].

### 4.5. Essential Oil

**Figure 8:**
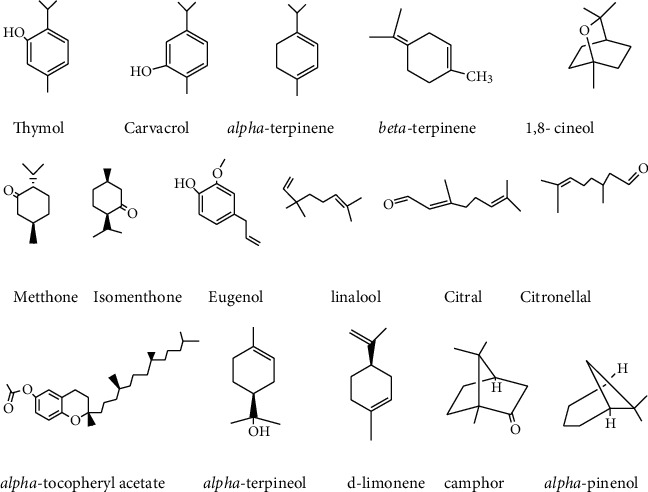
Anticancer components from different essential oil.

Essential oils (EOs) are well-known anticancer bioactive compounds obtained from medicinal and aromatic plants. Essential oils are highly volatile, aromatic yields obtained from plants. Due to their volatility, they can easily be extracted by steam distillation from different natural sources [111]. They may be a generic word for the liquid and highly volatile plant constituents with a distinct odor [111]. EOs having anticancer properties are listed in Figure 8. They are present in plants as secondary metabolites in their flowers, leaves, fruits, buds, seeds, rhizomes, barks, and roots [112, 113]. The essential oils such as limonene and perillyl alcohol, which is extracted from *Citrus sinensis*, are used for anticancer activities [114], and terpinene-4-ol, *α*-thujone, *β*-citronellal, *α*-pinene, *γ*-eudesmol, *δ*-cadinene, and methyl cinnamate from the Lamiaceae family are used for anticancer activities [115] as illustrated in Figure 8. The presence of bioactive compounds such as citronellyl acetate, pulegol, and citronellol in essential oils from *Pulicaria inuloides* was used for anticancer activity against liver, breast, and colorectal/colon cancers [116]. The essential oils derived from the flower of *Achillea ligustica*, leaf and the seed of *Coriandrum sativum*, leaf of *Melaleuca alternifolia*, the seed of *Nigella sativa*, and aerial parts of *Pelargonium graveolens* are used to treat different cancer diseases [117]. Some bioactive compounds such as linalool, 1,8-cineole, myrtenyl acetate, and myrtenol in *Myrtus communis* essential oil have anticancer properties in the case of blood cancer (leukemia) [83].

### 4.6. Other Bioactive Compounds

Various studies have shown that bioactive compounds such as fucoxanthin can be used to prevent breast cancer and triterpenes, anthocyanins, and saponins can be used to treat lung cancer. Blood cancer can be prevented using epigallocatechin gallate and rosavin [118]. Various compounds have been isolated from *Bersama abyssinica* to determine the plant's anticancer or antitumor function. Lignin and hallebergenin 3-acetate are two of these compounds that have been shown to inhibit tumor growth [22]. Garcinol, limonoids, crocin, and genistein are used to prevent pancreatic cancer [118]. The root of *India involucrate*, also known as “Yezngerotelba” in Amharic, can treat cancer, including diterpenes, and gnidicin, mezerein, gnidilatidin, gnidiglucin, and gniditrin are used to prevent cancer isolation biologically active compounds [22]. Boswellia acids in boswellia species give a defense mechanism to have anticancer activities [119]. The presence of gallic acid isolated from the leaves of *Kalanchoe petition*, which is commonly called “indahula,” is also essential to cure breast cancer [56]. It has been documented that the roots of *Asparagus africanus* are used to treat tumors [22]. Three lignans isolated from *Carissa spinarum*, namely, nortrachelogenin, carol, and carissanol, were found to be cytotoxic to WI38, MCF7, and A549 cell lines. Compared with carissanol and nortrachelogenin, carinol shows higher cytotoxic activity against these three cell lines, with an IC_50_ value of 1 *μ*g/mL [40].

## 5. Conclusion and Future Perspective

Several plant species are already being utilized to treat or prevent cancer. Multiple studies have identified plant species with anticancer characteristics, emphasizing herbal medicine in developing nations. In Ethiopia, many MPs can treat various types of cancer, such as breast cancer, lung cancer, blood cancer, and skin cancer and tumors. The ethnobotanical application of MPs for cancer treatment confirmed that plant leaves are the most valuable for preparing anticancer drugs (36.76%), followed by roots (27.2%), bark (12.5%), and flowers (1.5%). According to the analyzed data, the Euphorbiaceae family has the highest percentage (10.71%) of plant families utilized to treat cancer. The Asteraceae and Lamiaceae families have the second (7.1%) and third (6.1%) values, respectively. Regarding their habit, shrubs account for (39.5%) followed by herbs (33.8%), trees (17.9%), and climber or weed (8.8%).

Although numerous MPs have been utilized ethnobotanically to treat cancer, only a few MPs have been formally examined for anticancer activity. A few secondary metabolites and pure isolated compounds have been tested against cancer cell lines in vitro. Therefore, it is imperative to conduct detailed phytochemical research to isolate new anticancer drugs. Since the traditional knowledge for anticancer medicines provides basic information for further scientific research on the synthesis of anticancer drugs, it is necessary to conduct comprehensive ethnomedicinal research. The anticancer mechanism of these medicinal plant extracts is still unclear. Therefore, more in-depth scientific research is needed, which is the homework for researchers to conduct further studies.

## Figures and Tables

**Table 1:** Number of new cancer cases in 2020 in Ethiopia in number and percentage [34].

New cancer cases	Both sexes of all ages	Males of all ages	Females of all ages
Non-Hodgkin lymphoma	3,824 (4.9%)	2,359 (8.8%`)	1,465 (2.9%)
Leukemia	4,361 (5.6%)	2,565 (9.6%)	1,796 (3.55%)
Cervix uteri	7,455 (9.6%)	—	7,455 (14.7%)
Breast	16,133 (20.85%)	—	16,133 (31.9%)
Colorectum	6,048 (7.8%)	3,121 (11.7%)	2,927 (5.8%)
Prostate	2,720 (3.51%)	2,720 (10.2%)	—
Ovary	2,655 (3.43%)	—	2,655 (5.2%)
Other cancers	34,156 (44.15%)	15,989 (59.76%)	18,167 (35.9%)
Total	77,352	26,754	50,598

**Table 2:** Ethnobotanical uses of anticancer plants in Ethiopia.

Family	Habitat	Plant name	Local name	The region in which the plant can be found	Part of the plant used to treat cancer	Type of cancer	Ethnobotanical preparation	Reference
Acanthaceae	H	*Blepharis maderaspatensis*	Boke	Harari region	Roots	Breast cancer	The root is powdered and boiled with coffee used to like tea	[21]
Acanthaceae	S	*Justicia schimperiana*	Kitkit	North bench in SNR	Roots	Lung cancer	Until eating, fresh roots are crushed and boiled and the cool decoction is drank	[40]
Aloaceae	S	*Aloe pirottae*	It	Sidama zone in SNNP	Leaves	NSC	A spoonful of the plant's pulp or leaf is mixed with honey and consumed twice a day	[11]
Aloaceae	S	Aloe sp.	Gurtawaqota	Mizan Aman in SNMP	Leaves	NSC	The sap, which is made from the fresh root, is applied to the affected area	[30]
Amaranthaceae	H	*Aerva javanica*	Tobia	Dek Island in Amhara region	Roots	Breast cancer	The plant root is powdered and combined with the bat's blood and given orally before breakfast in the morning	[41]
Amaranthaceae	H	*Pupalia lappacea*	Kent-omme	Harari region	Leaves	NSC	The leaves are boiled and pasted with edible oil and orally taken	[21]
Amaranthaceae	W	*Achyranthes aspera*	Koch-ashite	Mizan Aman in SNMP	Leaves	NSC	Animal butter mixed with leaves of the plant roasted on a metal plate, crushed into powder, and then applied on the affected part	[30]
Amaryllidaceae	H	*Scadoxus multiflorus*	Dem-astefit	Mizan Aman in SNMP	Roots	NSC	Combination with other herbs and applied topically	[11]
Amaryllidaceae	H	*Crinum abyssinicum*	Yegibb shinkurt	Dek Island in Amhara region	Leaves	NSC	The leaf powder is administered topically to the afflicted area, together with hyena excrement and latex, to provide progressive relief	[42]
Anacardiaceae	T	*Ozora insignis*	Rukeylu, Garri	Harari region	Root	NSC	The plant's root has been crushed, and the bandage has been placed over it	[21]
Apiaceae	H	*Centella asiatica*	Goro-ngoc	Sheko in SNNPR	Leaves	NSC	The young leaves of the plant are powdered, and the sap is sniffed	[30]
Apiaceae	H	*Foeniculum vulgare*	Ensilal	East Gojjam in Amhara region	Roots	Lung cancer	The roots of the plant are mixed with other herbs used and taken orally	[30]
Apiaceae	H	*Hydrocotyle mannii*	Yeti-medhanit	North bench in SNNPR	Leaves	NSC	The leaves of the plant at a young age are powdered and put in the affected area	[30]
Apiaceae	H	*Ferula communis*	Dog	Libo-Kemkem in Amhara region	Root	Lung cancer	Fresh root crushed and drank with water orally	[43]
Apocynaceae	H	*Echidnopsis dammaniana*	Murali	Gene in Afar region	Stem	NSC	The stems of the plant are cut and the sap is introduced on the area of the affected part	[40]
Apocynaceae	H	*Catharanthus roseus*	Wulu-wusha	Dawro in SNNPR	Aerial part	NSC	Liver infection, wounds, and rheumatism	[44]
Apocynaceae	H	*Huernia macrocarpa*	Yemidirkulkual	In all the Amhara region	Latex	Skin cancer	The wound part is treated by inserting the mixture of the latex of the plant with “sumanfar”	[45]
Apocynaceae	S	*Carissa spinarum*	Agam	Bahir Dar Zuria in Amhara region	Twigs and leaves	Oral cancer	Honey mixed with a paste made by crushing the young twig and fresh leaf of the plant. The mixture is given orally until a cure	[46]
Apocynaceae	S	*Calotropis procera*	Kobo	Gene, in Afar region	Flower and root	NSC	The sap is added to the region after fresh roots are crushed	[47, 40]
Apocynaceae	C	*Pentarrhinum insipidum*	Barohula	Gewane in Afar region	Root	NSC	The plant's fresh roots are pounded, and the sap is added to the affected region	[30]
Asclepiadaceae	H	*Caralluma speciosa*	Ya'iibera	Harari region	Stem	NSC	The stem of the plant is crushed and bandaged in the affected part	[21]
Asclepiadaceae	S	*Kanahia laniflora*	August	Around West Gojjam in Amhara region	Leaves and latex	NSC	Fresh leaf juice with latex is taken orally or applied topically	[11]
Asparagaceae	C/W	*Asparagus africanus*	Sarita, hidden saree	Harari region in eastern Ethiopia	Root	Breast cancer	The concoction taken orally to treat tumors	[21]
Asparagaceae	C/W	*Asparagus africanus*	Yes-kest	Zegie Peninsula in Amhara regional state	Roots	Uterine cancer and breast cancer	The root is pounded, boiled, and drank	[48]
Asparagaceae	C/W	*Asparagus africanus*	Yes-kest	Kembatta Tembar in SNNPR	Root	Breast cancer	Used to treat uterine prolapse and breast tumours, among other things	[38]
Asphodelaceae	H	*Kniphofia foliosa*	Shushube	Bale Gobain Oromia region	Root	NSC	The dried roots are crushed and powdered and mixed with honey	[30]
Aspleniaceae	S	*Artemisia absinthium*	Ariti	None	Leaves	NSC	The leaves of the plant are mixed with *Zingiber officinale* and *Ruta chalepensis*, made into an infusion, filtered, and drank	[30]
Asteraceae	H	*Bidens macroptera*	Adey Abeba	Libo kemke district in Amhara region	Flower	Brain cancer	The powdered flower part is used	[43]
Asteraceae	H	*Bidens macroptera*	Adey Abeba	Libo Kemke in Amhara region	Leaves	NSC	The leaves are dried and powdered and applied to the area which needs to be cured	[43]
Asteraceae	H	*Artemisia absinthium*	Natura	Sodo Zuria in SNNP	Leaves	NSC	Dried leaves of the plant are powdered and macerated in coffee or tea	[30]
Asteraceae	H	*Artemisia afra*	Agfa	Doyo Gena in SNNPR	Leaves	NSC	Juice squeezed and taken orally	[30]
Asteraceae	H	*Cineraria abyssinica*	Item-firh	Bale Robe in Oromia region	Leaf and aerial parts	NSC	The aqueous decoction of the aerial and leaf parts of the plant is taken orally	[48]
Asteraceae	T	*Bacchae-oides filigera*	Weynagift	Nekem and Jimma in Oromia region	Leaves	NSC	Decocted leaf is drank to recover from lines	[30]
Asteraceae	H	*Artemisia annua*	Artemisia	Sodo Zuria in Sidama regional state	Leaves	NSC	Dried leaves are powdered and decocted in hot water and taken	[30]
Asteraceae	T	*Vernonia auriculifera*	Barawa	Doyo Gena	Leaves	Skin cancer	The leaves of the plant in a fresh state are grounded, and the sap is applied to it	[22]
Asteraceae	T	*Vernonia auriculifera*	Reji	Wondo Genet in SNNP	Leaves	Skin cancer	The plant's leaves in a fresh state are grounded, and the sap is applied to it	[22]
Asteraceae	S	*Echinops jericho*	Kericho	Harari region	Root	NSC	Powdered with *Hydnora johannis* tuber and added in the food that we eat	[21]
Asteraceae	H	*Guizotia scabra*	Sheshota	Doyo Gena in SNNPR	Leaves	NSC	The sap from fresh leaves is added to the affected area after they have been crushed	[30]
Asteraceae	S	*Vernonia auriculifera*	Barawa	SNMP	Leaves	NSC	The plant's fresh leaves are crushed, and the sap is added	[30]
Asteraceae	S	*Solanecio gigas*	Ababa	Doyo Gena in SNNPR	Leaves	NSC	The sap, made from fresh leaves of the plant, is crushed, and the sap is applied	[30]
Asteraceae	W	*Plectocephalus varians*	Este-Yohannes	Around West Gojjam in Amhara region	Whole part	NSC	The entire fresh plant is squeezed and applied	[11]
Asteraceae	S	*Vernonia amygdalina*	Grawa	Sidama regional state	Shoot	NSC	Tender shoots are pounded and soaked with water and given to the patient	[13]
Boraginaceae	T	*Cordia africana*	Size	South Gonder in Amhara region	Leaves	NS	The juice is made from the leaves of the plant and its paste is used to treat cancer	[46]
Boraginaceae	T	*Ehretia cymosa*	Makeba	Across the region of Ethiopia	Bark	Nectar and anal cancer	The root bark is applied topically in conjunction with other herbs	[11]
Brassicaceae	H	*Brassica carinata*	Gome-nzer	Debark district in Amhara region	Seed	Skin cancer	Seed of the plant with leaf and seed of the plant alone are crushed, powdered, and mixed with honey and creamed on the affected area	[49]
Capparidaceae	H	*Cleome brachycarpa*	Berbere	Gene in Afar regional state	Leaves	NSC	Fresh leaves of the plant are grounded, and the sap is placed on the affected part	[30]
Capparidaceae	T	*Boscia senegalensis*	None	None	Root	NSC	By pounding and powdering given orally	[50]
Capparidaceae	S	*Canada farinosa*	Qala-anqaal	Yalo district, zone 4 in Afar region	Leaves	Breast cancer	Not found	[51]
Celastraceae	S/T	*Maytenus senegalensis*	Kebkeb	Gondar Zuria district in Amhara region	Bark	NSC	The plant's bark is crushed, boiled, and filtered, and one cup is served	[52]
Celastraceae	S	*Maytenus ovatus*	Not specified	NA	Leaf	NSC	The plant's leaf paste, mixed with honey, is taken orally every morning and evening before it heals	[53]
Celastraceae	S	*Gymnosporia buchanan*	Atat	Gondar in Amhara region	Leaves	NSC	Crushed leaves are mixed with honey to produce a paste	[54]
Celastraceae	S	*Maytenus senegalensis*	Atat	Gondar Zuria district, Amhara region	Leaves	NSC	It is applied to the affected region with a paste	[52]
Combretaceae	T	*Lagenaria siceraria*	Basubaaqula	Dega Damot district/Amhara region	Fruit	Breast cancer	The leaves of the plant are powdered, squeezed, and put on the affected area (wound)	[55]
Combretaceae	T	*Combretum collinum*	Abalo	Debark district, North Gondar zone in Amhara region	Leaves	NSC	The leaves of the plant are grounded, crushed, and put on the wound or tumour	[11]
Commelinaceae	H	*Commelina benghalensis*	Value-cha	Doyo Gena in SNMP	Roots	NSC	Fresh roots of the plant are dried and pounded, and the sap is put on the affected part	[30]
Convolvulaceae	S	*Ipomoea marmorata*	Gumna-kul	Harari region in eastern Ethiopia	Root	NSC	The new tuber is consumed, and a concoction is taken orally	[21]
Crassulaceae	H	*Kalanchoe petition*	Inda-hula	Bale in Oromia region	Leaves	Breast cancer and skin cancer	The plant leaves, fresh, are soaked for two minutes and put on the affected part. The plant is powder and mixed with hyena faces and latex	[22]
Crassulaceae	H	*Kalanchoe lanceolata*	Bose	Nekemte in Oromia region	Leaves and roots	NSC	The juice which is made from the fresh root and leaves is squeezed and drank	[56]
Cucurbitaceae	H	*Cucumis prophetarum*	Yemdirembuay	Debre Libano in Oromia region	Roots	Skin cancer	The root of the plant is dried and powdered and, when combined with water, given orally	[42]
Cucurbitaceae	H	*Clutia abyssinica*	File-fej	Across the region of Ethiopia	Whole part	NSC	The whole part of the plant, together with *Coffea robusta* and *Coffea richardiana*, is used topically	[11]
Cucurbitaceae	H	*Momordica friesiorum*	Wof tech	Across the region of Ethiopia	Roots	NSC	The root of the plant is combined with other herbs and applied topically	[11]
Cucurbitaceae	T	*Croton macrostachyus*	Bisana	Hawassa, Sidama regional state	Leaves	Skin cancer and wound cancer	The juice of the leaves of the plant and its paste are applied on wound cancer, and crushed and powdered fresh leaves are used on the affected part	[46, 30]
Cucurbitaceae	W/C	*Lagenaria siceraria*	Qil	Hawassa city/Sidama regional state	Root	NSC	The root of the plant is pounded, powdered, and drank	[39]
Cucurbitaceae	W/C	*Lagenaria siceraria*	Qil	None	Leaves	NSC	Crushed and squeezed leaves are applied to the infected area to alleviate cancerous sores	[57]
Euphorbiaceae	T	*Euphorbia tirucalli*	Kinc hib	South Wollo in Amhara regional state	Latex of roots	Skin cancer	The fresh sap/latex of the plant is collected and creamed all over the body. Latex is given for topical application	[58]
Euphorbiaceae	H	*Euphorbia platyphyllos*	Anitrfa	Mecha district in Amhara region	Latex	NSC	Fresh latex of the plant is put topically on the tumour	[22]
Euphorbiaceae	H	*Euphorbia lathyris*	Hada-amii	Chelya district in Oromia region	Stem	Breast cancer	Steam of the plant is chopped and fumigated to the affected breast	[22]
Euphorbiaceae	T	*Euphorbia abyssinica*	Cultural	Around Debre Libanos in Oromia region	Latex	Skin cancer	Latex is eaten with teff powder of the plant bread or takes the latex and then painted on the spot	[45]
Euphorbiaceae	T	*Acacia oerfota*	Seraw	Yalo district zone 4 in Arar region	Leaves	Breast cancer	The leaf of the plant is crushed and put nasally and topically	[11]
Euphorbiaceae	T	*Euphorbia abyssinica*	Qulqwal	Debre Libanos in Oromia region	Latex, stem, and bark	Skin cancer	Decoction and placing of the latex to the affected part; and the paste of the bark and stem is rub to the affected area	[42]
Euphorbiaceae	T	*Euphorbia tirucalli*	Kinship	Dale district in Sidama regional state	Bark	Skin cancer	Latex is combined with powder made from beans given to eat after food, and latex is dropped on the affected part to treat skin cancer	[58]
Euphorbiaceae	T	*Euphorbia tirucalli*	Kinship	Fiche in Oromia region	Latex and root	Skin and neck cancers	Eaten and added to the skin after being mixed with bean powder	[59]
Euphorbiaceae	T	*Erythrina brucei*	Kiara/Woolens	Dale district in Sidama region	Bark	NSC	The juice made from the bark of the plant is drank for the treatment of cancer	[46]
Euphorbiaceae	T	*Croton macrostachyus*	Bisana	Hawass in Sidama region	Leaves and seeds	NSC	The leaves or the seed of the plant are crushed and inserted into the wound	[45]
Euphorbiaceae	S	*Ricinus communis*	Qenbo'o	Hawassa in Sidama region	Root	Breast cancer	The root of the plant is chewed and swallowed or applied to the affected part	[30]
Euphorbiaceae	S	*Colutea abyssinica*	Graduate	Across the region of Ethiopia	Root and seed	Cervical and rectal cancer	The root and seed are mixed with other herbs and given topically	[11]
Euphorbiaceae	S	*Jatropha curcas*	Ayderke	NA	Seed	NSC	Tumours are treated with a paste made from the plant seed powder mixed with honey	[44]
Euphorbiaceae	T	*Acalypha acrogyna*	Gullo	Gondar in Amhara region	Leaves	NSC	The leaves of the plant are crushed and combined with honey	[60]
Euphorbiaceae	T	*Acalypha acrogyna*	Gullo	Gondar in Amhara region	Leaves	NSC	In the morning, a mixture of honey and paste made from the leaves of the plant is given orally and heated leaves are applied externally over the affected area	[42]
Euphorbiaceae	S	*Senna alexandrina*	Mekerbaa	NA	Bark	NSC	The powdered bark of the plant is creamed on the swelling	[11]
Euphorbiaceae	S	*Euphorbia schimperiana*	Gendal-elata	Doyo Gena in SNNPR	Root	NSC	The plant's fresh roots are crushed, and the sap is added to the affected area	[13]
Euphorbiaceae	S	*Euphorbia polyacantha*	Carrico	NA	Latex	Skin cancer	The latex of the plant is squeezed and creamed on the affected part	[42]
Euphorbiaceae	S	*Calpurnia aurea*	Digita	Debre Libanos monastery in Oromia region	Leaves	Neck cancer	The leaves of the plant are powdered and soaked in cold water and taken orally	[22, 42]
Euphorbiaceae	S	*Senna singueana*	Busha	Across the region	Leaves and bark	NSC	The powdered leaves of the plant are applied topically	[30]
Euphorbiaceae	S	*Dichrostachys cinerea*	Ader	Yalo district in Afar region	Root	Skin cancer	The root of the plant is pounded and given orally	[51]
Fabaceae	T	*Acacia seyal*	Wacho	Bensa in SNMP	Leaves	NSC	The leaves of the plant are chewed and swallowed	[39]
Fabaceae	T	*Albizia lebbeck*	NA	Adekfurdu in Tigray region	Root	NSC	Wheat dough paste of root powder is applied on the affected part	[61]
Fabaceae	S	*Melilotus suaveolens*	Egg	Gubalafto district in northern Ethiopia	Leaves	Lung cancer	Crush, smash in water, filter, and then drink	[45]
Fabaceae	S	*Calpurnia aurea*	Vegeta	Debre Libanos in Oromia region	Leaves	Neck cancer	Powder is mixed with water and taken orally	[59]
Fabaceae	S	*Calpurnia aurea*	Digita	Debre Libanos in Oromia region	Leaves	Neck cancer	The leaves of the plant are made a paste and put on the affected area	[22]
Fabaceae	S	*Calpurnia aurea*	Digita	Bahir Dar Zuria in Amhara region	Leaves and seed	NSC	Powdered leaves or seeds are immersed in cold water and then drank	[42]
Fabaceae	S	*Crotalaria incana*	Chelke	Doyo Gena in SNNPR	Leaves	NSC	Fresh leaves are pounded and the sap was put on the affected area	[30]
Fabaceae	S	*Senna singueana*	Gefa	Bahir Dar Zuria in Amhara region	Leaves	NSC	Fresh leaves are pounded, soaked in water, and drank	[30]
Fabaceae	S	*Crotalaria agatiflora*	Unknown	Bale Goba in Oromia region	Seed	NSC	Dry seeds are powdered, mixed with honey, and put on the affected area	[30]
Fabaceae	T	*Millettia ferruginea*	Henge-ddicho	Sidama regional state	Bark	NSC	The juice of bark is drank for cancer treatment	[46]
*Flacourtiaceae*	S	*Dovyalis abyssinica*	Kashim	Fiche district in Oromia region	Fruit	NSC	Eating 6–10 fruits per day	[58]
*Flacourtiaceae*	S	*Dovyalis abyssinica*	Kashim	Dale district in SNMP	Bark	NSC	The raw bark of the plant was chewed and then consumed	[58]
Hydnoraceae	T	*Hydra abyssinica*	Shifa'-a weyn	Harari region	Bark and roots	NSC	Bark or root of the plant is powdered with *Echinopskebericho* tuber and added in the daily food and eating	[21]
Iridaceae	H	*Gladiolus candidus*	Milas-golgul	Dega Damon in Amhara region	Roots	NSC	The root is dried and powdered and put on the affected area or drank	[57]
Iridaceae	H	*Gladiolus candidus*	Milas-golgul	Dega Damot in Amhara region	Roots	NSC	The plant's root is dried, crushed, and put on the wound part, or root powder is taken orally with water	[57]
Juncaceae	H	*Juncus effusus*	Etse-felatsut	Across the region of Ethiopia	Roots	NSC	The root of the plant is used by mixing with other herbal plants and applied topically on the affected area	[11]
Juncaceae	H	*Cleroden-drum myricoides*	Misrichi	Dale district in SNNP	Leaves	Blood cancer	The honey is mixed with the grounded leaf part of the plant and drank, or the root of the plant is boiled and mixed with Z*anthoxylum chalybeum* shoot	[49]
Lamiaceae	H	*Leonotis ocymifolia*	Arma-USA	Bale Goba in Oromia region	Leaves	NSC	Fresh leaves are crushed, macerated overnight, and drank	[30]
Lamiaceae	H	*Ajuga leucantha*	Tiksasht	North Bench in SNMP	Leaves	NSC	The fresh leaves of the plant are grounded, and the sap is put on the affected area	[30]
Lamiaceae	H	*Ocimum gratissimum*	Make-desisa	Wendo Genet in SNMP	Roots	NSC	Fresh roots are crushed, boiled, and drank	[30]
Lamiaceae	H	*Salvia nilotica*	Keskeo	North Bench in SNNP and Gonde in Amhara region	Leaves	NSC	The fresh leaves of the plant are powdered with water and made a paste	[60]
Lamiaceae	H	*Thymus schimperi*	Design	Bale Goba in Oromia region	Leaves	NSC	Dry leaves are decocted and drank	[30]
Lamiaceae	S	*Premna schimperi*	Xullangee	Bule Horra in Oromia region	Leaves	NSC	Pounding and making solution	[5]
Lamiaceae	T	*Pycnostachys abyssinica*	Montana	Doyo Gena in SNNPR	Leaves	NSC	The sap is added to the affected area by crushing the pounded fresh leaves of the plant	[30]
Lamiaceae	S	*Leonotis ocymifolia*	Ye fereszeng	Fiche district in Oromia region	Leaves	Neck cancer	For one day, the chopped leaves of the plant are applied to the affected area	[62]
Lamiaceae	S	*Leonotis ocymifolia*	Raskimir	Across the region of Ethiopia	Root	NSC	Sometimes, it is used with the combination of *Leonotis africana*	[11]
Lamiaceae	S	*Satureja abyssinica*	Este meaza	Across the region of Ethiopia	Leaves	NSC	The leaves of the plant are combined with other herbs and applied topically	[11]
Lamiaceae	S	*Roca myricoides*	Mardhisiis a	Bule Hora, Oromia region, Bensa in SNMP	Leaves and root	NSC	Crush the root, mix it with butter, and apply; chop the leaf and eat or apply	[30, 49]
Liliaceae	H	*Gloriosa superba*	Etse-lebona	In most of Ethiopia and the Harari region	Roots, seeds, and leaves	Breast cancer	Seeds and roots of the plant dried and crushed and mixed with water are taken orally. The root of the plant is chewed and put externally on the affected breast. The leaves of the plant are made paste and tied on the tumour	[21, 42]
Lobeliaceae	T	*Lobelia rhynchopetalum*	Etse-kemun	Across the region of the country	Root	NSC	The root of the plant is combined with other herbs and put topically	[45]
Loganiaceae	S	*Buddleja polystichum*	Anfar	Dale district in SNNP	Leaf	NSC	Crushed, cold macerated, and taken orally	[49]
Loganiaceae	W/C	*Malva verticillata*	Lut	Ada's district, east Shewa zone in Oromia region	Leaves	NSC	The leaf is crushed and attached to the swelling after being warmed over an open flame	[13]
Malvaceae	S	*Sida schimperiana*	Kotejebessa	Wendo Genet in SNMP	Root and leaves	Wound cancer	Fresh leaves and roots of the plant are pounded, macerated, and drank	[30]
Malvaceae	S	*Sida schimperiana*	Chef Greg	Nekemte town, east Wellega in Oromia region	Root	Breast cancer	The juice made from fresh root is mixed with honey and taken orally	[30]
Malvaceae	S	*Sida schimperiana*	Chef Greg	Debark district in Amhara region	Leaves and root	Neck cancer	The root and leaves of the plant are crushed, powdered, and then put on the affected part	[11]
Malvaceae	H	*Malva verticillata*	Lut	East Shewa zone in Oromia region	Leaves	Neck cancer	The leaf of the plant is crushed, warmed, and then tied on the swelling	[13]
Meliaceae	T	*Lepidotrichilia volkensii*	Tabecho	Bensa in SNNP	Leaves and fruit	NSC	The leaves and fruit of the plant are chopped and mixed with water and taken orally	[63]
Melianthaceae	T	*Bersama abyssinica*	Azamirr	Bahir Dar Zuria in Amhara region	Bark	NSC	The plant's bark or stem is used to make an injection used to treat some types of tumours	[22]
Melianthaceae	T	*Bersama abyssinica*	Azamir	Dale district in Sidama region	Bark	NSC	The bark of the plant is crushed and boiled, and then a small amount is drank	[30]
Menispermaceae	S/C	*Stephania abyssinica*	Kalala	Nekemte in Oromia region	Roots	Skin cancer	Honey is mixed with the juice prepared from the root of the plant and taken to give relief	[11]
Menispermaceae	S/C	*Stephania abyssinica*	Hidden	Harari region	Roots	NSC	The root of the plant is dried and crushed and pasted and bandaged on the affected area	[21]
Menispermaceae	S/C	*Stephania abyssinica*	Kalala	Wondo genet in SNNP	Leaves	Skin cancer	Fresh leaves of the plant are massaged by hand, and droplets are applied to the affected area	[22]
Menispermaceae	S/C	*Stephani abyssinica*	Yeayethareg	Across the region of Ethiopia	Roots	NSC	The leaf of the plant is boiled, and about one cup is drank for a treatment	[13]
Moraceae	H	*Dorstenia barnimiana*	Work-bemeda	Dekisland in Amhara region and Harari region	Roots, tuber, and aerial parts	Hemorrhoid cancer	Aerial parts of the plant are powdered and made paste with butter and put on the top part of the affected area. To treat the affected area, fresh or dry root is inserted in the opening part	[21, 64]
Moraceae	H	*Dorstenia barnimiana*	Work bemeda	Around Bahir Dar Zuria Woreda in Amhara region	Roots	NSC	The roots of the plant are dried and grounded and mixed with honey and water and drank. Inserting fresh dry root at the affected part	[59]
Moraceae	S	*Dorstenia barnimiana*	Worqbemeda	Bahir Dar Zuria in Amhara region	Root	NSC	Fresh roots of the plant are then crushed and applied	[21, 11]
Myrsinaceae	S	*Myrsine africana*	Quechee	Fiche district in Oromia region	Fruit	NSC	Dried fruit and leaves of plant are powdered and mixed with little water and taken orally	[58]
Phytolaccaceae	S	*Phytolacca dodecandra*	Endod	Bensa and Dawro in SNMP	Leaves and root	NSC	The leaves of the plant are chopped or pounded and applied to the affected part	[11]
Pittosporaceae	S	*Pittosporum abyssinicum*	Lola	Dale district in SNNP	Bark	NSC	The juice made from the bark of the plant is drank for the treatment of cancer	[42]
Plumbaginaceae	S	*Plumbago zeylanica*	Amera	Bahir Dar Zuria in Amhara region	Roots, leaves, and bark	NSC	Powdered together with onion and honey	[65]
Plantaginaceae	H	*Plantago lanceolata*	Yebeglat	Hawassa city in SNNPR	Seed	NSC	The seed of the plant is crushed, powdered, and applied to the affected area	[11]
Plantaginaceae	H	*Plantago lanceolata*	Gorteb	Sidama regional state	Seed	NSC	The dried seeds are powdered, crushed, and put into the cancer wound	[39]
Plumbaginaceae	S	*Plumbago zeylanica*	Amera	Harari region	Roots	Bone cancer	The root is powdered and pasted on the affected area and bandaged	[21]
Plumbaginaceae	S	*Plumbago zeylanica*	Amira	Tigray, Amhara, Oromia region	Roots	NSC	The root of the plant is powder and combined with sulphur and placed on top position or powdered and drank with boiled tea or coffee	[65, 51]
Plumbaginaceae	S	*Plumbago zeylanica*	America	Bahir Dar Zuria in Amhara region	Leaves	NSC	The juice is made from fresh leaves and taken orally	[42]
Podocarpaceae	T	*Podocarpus falcatus*	Bribie	Not specified	Root	NSC	The plant's dry root powder is mixed with water and is taken orally and applied topically to the affected area	[66]
Podocarpaceae	T	*Afrocarpus falcatus*	Zigba	Dale district in Sidamo region	Leaves	NSC	The juice of the leaf is taken for treating cancer	[46]
Podocarpaceae	T	*Afrocarpus falcatus*	Zigba	Dek Island in Amhara region	Root	NSC	Powdered dry root combined with water	[46]
Polygonaceae	H	*Rumex abyssinicus*	Mekumoko	Harari region	Rhizome	Breast cancer	Decocted hot infusion is taken orally	[21]
Polygonaceae	H	*Rumex abyssinicus*	Moke-moko	Seharti Samre in Tigray region	Roots	NSC	The root of the plant is powdered and mixed in a spicy stew and then used	[67]
Polygonaceae	H	*Rumex abyssinicus*	Mem-eqo	Across the region of Ethiopia	Roots	Breast cancer	The root of the plant is powdered and creamed on the affected area of swelling. Decocted hot infusion is taken orally	[21, 11]
Polygonaceae	H	*Rumex nepalensis*	Groucho	Doyo Gena in SNMP	Roots and bark	NSC	The dried roots of the plant are crushed and given with food, or the sap from the fresh bark is crushed and squeezed and then put on the affected area	[43]
Polygonaceae	H	*Rumex nervosus*	Huot/Embuacho	Seharti Samre district in Tigray region	Leaf	Breast cancer	The leaves of the plant are pounded, and its paste is put on the affected area	[67]
Polygonaceae	H	*Rumex nervosus*	Huot	Seharti Samre in Tigray region	Leaves	NSC	Leaves are crushed and the paste is applied on the affected area	[67]
Punicaceae	T	*Punica granatum*	Roman	Libo Kemke in Amhara region	Fruit	NSC	Crushed the fruit of the plant and eaten	[21]
Ranunculaceae	H	*Ranunculus multifidus*	Etsesiol	Debre Libanos monastery in Oromia region	Roots	NSC	On the affected area, the paste of the root is applied externally	[42]
Ranunculaceae	H	*Delphinium dasycaulon*	Sam-onya	Harari region	Root	NSC	Concoction is taken orally	[21]
Ranunculaceae	H	*Thalictrum rhynchocarpum*	Sire Bizu	Across the region of Ethiopia	Roots	NSC	The root of the plant is mixed with other herbs and put topically	[11]
Ranunculaceae	H	*Ranunculus multifidus*	Etsesiol	Debre Libanos monastery in Oromia region	Leaves	NSC	Externally, the affected area is covered by the powdered leaves of the plant	[59]
Ranunculaceae	H	*Ranunculus multifidus*	Etsesiol	Debre Libanos monastery in Oromia region	Roots	NSC	Powder dressing on the affected area	[59]
Ranunculaceae	S/C	*Clematis hirsuta*	Amazon-hareg	All over Ethiopia	Leaves, stems, and bark	Neck cancer	The plant's bark, leaves, and stems are grounded into a powder that is applied directly on tumour sites	[42]
Ranunculaceae	W/C	*Clematis simensis*	Yeazo-hareg	Libo Kemke/Amhara region	Leaves	NSC	Crushed and applied	[45]
Ranunculaceae	C	*Clematis virginiana*	Fidy	Bale/Oromia region	Leaves	NSC	Pounding the leaves, making solution, or mixing with butter	[30]
Ranunculaceae	C	*Clematis simensis*	Fireside	Libo Kemkem district/Amhara region	Leaves	Skin cancer	Crush the leaf and add it to the infected area	[42]
Ranunculaceae	C/S	*Clematis hirsuta*	Amazon-hareg	Bale/Oromia region	Leaves, stem, and bark	Neck cancer	The leaves are crushed and used as a bandage on the swelling	[57]
Ranunculaceae	C/S	*Clematis hirsuta*	Amazon-hareg	All over Ethiopia	Leaves, stems, and bark	Neck cancer	The plant's bark, leaves, and stems are grounded into powder that is applied directly on tumour sites	[42]
Ranunculaceae	C	*Clematis simensis*	Yeazo-hareg	Libo Kemke/Amhara region	Leaves	NSC	Crushed and applied	[45]
Ranunculaceae	C/S	*Clematis hirsuta*	Amazon-hareg	Bale/Oromia region	Leaves, stem, and bark	Neck cancer	The leaves are crushed and used as a bandage on the swelling	[57]
Rhamnaceae	T	*Ziziphus spina-christi*	Geba	All over Ethiopia	The whole parts	NSC	It is used for the treatment of tumour	[42]
Rosaceae	T	*Prunus africana*	Tikurenchet	Bensa in SNNP	Bark and leaves	NSC	Powdered bark of the plant is applied on the skin of the patient to get relief	[68]
Rosaceae	T	*Hagenia abyssinica*	Kosso	Across the regions of Ethiopia	Root	NSC	Honey is mixed to the pounded root of the plant and then creamed on the affected part	[11]
Rosaceae	T	*Prunus africana*	Tikurenchet	Bensa, SNNP	Powdered bark	NSC	Swelling the powdered bark of the plant	[68]
Rubiaceae	C	*Rubia cordifolia*	Enchibir	Gubalafto district in Amhara region	Roots	Lung cancer	The root part of the plant is powdered in water for three days and given orally	[38]
Rubiaceae	C	*Rubia cordifolia*	Enchibir	Across the regions of Ethiopia	Roots	NSC	The root of the plant mixed with other herbs and put topically on the affected area	[11]
Rubiaceae	T	*Pavetta gardeniifolia*	Qadiidaa	Bule Horra in Oromia region	Root	NSC	Pounded and applied	[69]
Rutaceae	T	*Zanthoxylum chalybeum*	Gada	Hawass in SNNR	Leaves	NSC	The leaves of the plant are powdered and drank	[69]
Rutaceae	T	*Fagaropsis angolensis*	Dergi	NA	Fruit	NSC	The juice made from the fruit of the plant is taken orally and applied externally to the affected area	[42]
Rutaceae	S	*Clausena anisata*	Limit	Abay Gorge/Amhara region	Leaves	NSC	Dry leaves of the plant are powdered and mixed with honey and eaten	[30]
Santalaceae	T/S	*Osyris quadripartita*	Quote	Around Fiche district in Oromia region	Leaves	NSC	The dried leaf of the plant is mixed with dried and grounded fruit of Myrsine africana and combined with water and taken orally	[59]
Sapindaceae	T	*Dodonaea viscosa*	Kitkita	Bahir Dar Zuria in Amhara region	Root	NSC	Honey is mixed with the dried and powdered roots of the plant and drank	[46]
Sapindaceae	T	*Dodonaea angustifolia*	Ketketa	Wide range part in Ethiopia	All parts	Neck cancer	The paste, which is made from whole parts of the plant is put on the affected area	[18]
Sapotaceae	T	*Mimusops kummel*	Safa	Berber district in Oromia region	Root	Lung cancer	The root and fruit of the plant are grounded and dissolved with a small amount of water and taken orally to treat lung cancer	[6]
Sapotaceae	T	*Mimusops kummel*	Galati	Berbere district in Oromia region	Root	Lung cancer	The roots are powdered, and a small amount is ingested with water	[6]
Sapotaceae	T	*Mimusops kummel*	Ishe	Benishangul, Amhara, and Gambela region	Fruit and root	Lung cancer	The root and fruits of the plant are grounded and dissolved with a small amount of water and taken orally	[6]
Sapotaceae	S	*Sideroxylon oxyacanthum*	Bunguude	Dalle district in Sidama region	Leaves	Cancer	The leaf is macerated and given an overall flavour, sometimes with Zanthoxylum chalybeum leaf and honey	[49]
Scrophulariaceae	S	*Verbascumsinaiticum*	Yefereszeng	Dek Island in Amhara region	Roots	Breast cancer	Powder mixed with hyena feces and latex	[22, 42]
Simaroubaceae	T	*Brucea antidysenterica*	Abalo	Jimma in Oromia region	Leaves	NSC	The leaves of the plant are powdered and mixed with young twigs to make pastes and placed on the affected area	[11]
Simaroubaceae	T	*Brucea antidysenterica*	Abalo	Jimma in Oromia region	Steam, bark, and leaves	NSC	Paste is made from leaves and young twigs with water and drank before meals	[42]; [30]
Simaroubaceae	T	*Brucea antidysenterica*	Waginos/Apollo	Jimma zone and Bale zone in Oromia region	Steam, bark, and leaves	NSC	The decoction is drank, and pastes are made from young twigs and powered leaves with water	[13]
Solanaceae	S	*Discopodium penninervium*	Chechanga	Doyo Gena in SNNPR	Leaves	NSC	Fresh leaves of the plant are crushed and applied on the affected area	[30]
Solanaceae	S	*Solanum nigrum*	Embuayzerech	Across the region of Ethiopia	Leaves, stems, and roots	NSC	The herb is boiled and put in our food daily for about three days	[22]
Solanaceae	S	*Withania somnifera*	Ozawa	NA	Root	NSC	The root is directly chewed orally	[11]
Solanaceae	S	*Lycopersicon esculentum*	Tematim	All over Ethiopia	Fruit	NSC	Without cooking, fresh fruit is washed and ate	[42]
Solanaceae	H	*Solanum americanum*	Tikurawut	NA	Leaves, root, and steam	NSC	Leaves are boiled thoroughly and eaten	[67]
Thymelaeaceae	H	*India involucrata*	Yezinge-rotelba	NA	Roots	NSC	The root of the plant is powdered and made paste with honey	[30]
Verbenaceae	S	*Lantana trifolia*	Hanshi-Bello	Wondo Genet in SNMP	Leaves	NSC	Fresh leaves are powdered and drank after being immersed in cold spring water	[30]
Verbenaceae	S	*Lippia adoensis*	Kessie	Abay gorge in Amhara region	Leaves	NSC	The dried leaves are powdered, soaked in cold water, and drank	[30]
Vitaceae	C	*Rhoicissus tridentate*	Buriguraa	Harari region	Root	NSC	Concoction is taken orally	[21]
Vitaceae	C	*Cyphostemma serpens*	Eirini	Gewan/Afar region	Root	NSC	Dry roots are grounded, then eaten, and added after being pasted with honey	[30]
Zygophyllaceae	H	*Tribulus terrestris*	Camera	Across the regions of Ethiopia	All parts	NSC	The plant is recommended as an anticancer	[70]

NSC: nonspecified cancer; H: herb; S: shrub; T: tree; W: weed; C: climbing plant.

## Abbreviations

AIF: Alpinumisoflavone
FAA: Flavone-8-acetic acid
MLF: 4′-Methoxylicoflavanone
MP: Medicinal plant
MTT: 3-(4,5-Dimethylthiazol-2-yl)-2,5-diphenyl-2H-tetrazolium
SRB assay: Sulforhodamine B
EOs: Essential oil
NSC: Nonspecified cancer
SNNP: Southern Nations, Nationalities, and peoples
TMs: Traditional medicines
WHO: World Health Organization.
